# Quantitative Soil Characterization for Biochar–Cd Adsorption: Machine Learning Prediction Models for Cd Transformation and Immobilization

**DOI:** 10.3390/toxics12080535

**Published:** 2024-07-24

**Authors:** Muhammad Saqib Rashid, Yanhong Wang, Yilong Yin, Balal Yousaf, Shaojun Jiang, Adeel Feroz Mirza, Bing Chen, Xiang Li, Zhongzhen Liu

**Affiliations:** 1 Key Laboratory of Plant Nutrition and Fertilizer in South Region, Ministry of Agriculture, Guangdong Key Laboratory of Nutrient Cycling and Farmland Conservation, Institute of Agricultural Resources and Environment, Guangdong Academy of Agricultural Sciences, Guangzhou 510640, China; saqibssr@mail.ustc.edu.cn (M.S.R.); wangyanhong@gdaas.cn (Y.W.); yilong516@163.com (Y.Y.); shaojunj93@163.com (S.J.); 2 Department of Technologies and Installations for Waste Management, Faculty of Energy and Environmental Engineering, Silesian University of Technology, 44-100 Gliwice, Poland; balal.yousaf@polsl.pl; 3 Department of Mechanical and Energy Engineering, Southern University of Science and Technology, Shenzhen 518055, China; adeelmirza@mail.ustc.edu.cn; 4 Key Laboratory of Animal Nutrition and Feed Science in South China, Guangdong Provincial Key Laboratory of Animal Breeding and Nutrition, Collaborative Innovation Center of Aquatic Sciences, Institute of Animal Science, Guangdong Academy of Agricultural Sciences, Ministry of Agriculture and Rural Affairs, Guangzhou 510640, China; chenbing114@163.com

**Keywords:** biochar, cadmium, transformation, remediation, prediction models

## Abstract

Soil pollution with cadmium (Cd) poses serious health and environmental consequences. The study investigated the incubation of several soil samples and conducted quantitative soil characterization to assess the influence of biochar (BC) on Cd adsorption. The aim was to develop predictive models for Cd concentrations using statistical and modeling approaches dependent on soil characteristics. The potential risk linked to the transformation and immobilization of Cd adsorption by BC in the soil could be conservatively assessed by pH, clay, cation exchange capacity, organic carbon, and electrical conductivity. In this study, Long Short-Term Memory (LSTM), Bidirectional Gated Recurrent Unit (BiGRU), and 5-layer CNN Convolutional Neural Networks (CNNs) were applied for risk assessments to establish a framework for evaluating Cd risk in BC amended soils to predict Cd transformation. In the case of control soils (CK), the BiGRU model showed commendable performance, with an $R^{2}$ value of 0.85, indicating an approximate 85.37% variance in the actual Cd. The LSTM model, which incorporates sequence data, produced less accurate results ($R^{2}=0.84)$, while the 5-layer CNN model had an *R*^2^ value of 0.91, indicating that the CNN model could account for over 91% of the variation in actual Cd levels. In the case of BC-applied soils, the BiGRU model demonstrated a strong correlation between predicted and actual values with $R^{2}$ (0.93), indicating that the model explained 93.21% of the variance in Cd concentrations. Similarly, the LSTM model showed a notable increase in performance with BC-treated soil data. The $R^{2}$ value for this model stands at a robust $R^{2}\;(0.94)$, reflecting its enhanced ability to predict Cd levels with BC incorporation. Outperforming both recurrent models, the 5-layer CNN model attained the highest precision with an $R^{2}$ value of 0.95, suggesting that 95.58% of the variance in the actual Cd data can be explained by the CNN model’s predictions in BC-amended soils. Consequently, this study suggests developing ecological soil remediation strategies that can effectively manage heavy metal pollution in soils for environmental sustainability.

## 1. Introduction

Natural and synthetic processes release heavy metals (HMs) into the environment, including volcanic eruptions, weathering, wastewater irrigation, sewage sludge disposal, smelting, and pesticide application [1,2]. Furthermore, ingestion and inhalation absorption are the main routes by which HMs can accumulate in the human body [3]. The soil–crop system provides an additional route by which HMs can accumulate in humans [4,5,6]. In organisms, Cd can accumulate for 50 years, and its half-life is 10 to 30 years. According to a 2019 Environmental Protection Agency (EPA) report, Cd and its compounds were considered hazardous metals and toxic water pollutants [7]. Approximately 7.0% of the soils of China was found to contain excess Cd, which ranked first among inorganic pollutants [8].

The physiochemical composition and texture of soil determine its quality and production [9]. These characteristics form the foundation of the natural environment and crops [10]. HM pollution is a significant by-product of industrialization in various countries worldwide [11]. China’s rapid industrialization has inevitably produced similar problems, and governments at all levels are committed to remediating HM-contaminated soils [12]. In addition to microbial activity, organic matter can contribute to the immobilization of Cd through precipitation and complexation processes in soil [13,14]. Standard methods used for HM analysis include ultraviolet-visible spectrophotometry, gas chromatography–mass spectrometry, inductively coupled plasma emission spectrometry, and atomic fluorescence spectrometry [15]. While these technologies possess high sensitivity and accuracy, their short detection range, labor-intensive and time-consuming detection techniques, and complex preparation of samples contribute to their being unsuitable for quick, non-intrusive, and batching testing [16,17]. 

In recent years, artificial intelligence (AI) deep learning approaches have conquered the limitations of conventional diagnosing methods [18]. Such approaches improve efficiency and adaptability by reducing the amount of initial processing and incorporating the required extract [19]. Deep learning is a hierarchical structure that uses machine learning techniques to identify and collect significant characteristics [20]. It improves its performance via training, resulting in high precision. Statistical and CNN approaches estimate tangible qualities from the input [21]. Among the emerging soil amendment materials in recent years, BC has been regarded as one of the most effective [22]. Several studies have demonstrated that BC, a charcoal derived from organic matter, is highly effective as an adsorbent for Cd in soil [23]. It has been found that clay minerals and organic matter in soil can enhance BC’s performance in the adsorption of Cd [24]. Consequently, clay minerals and organic matter increase the soil’s surface area. This leads to an increase in the surface area accessible for the adsorption of Cd and facilitates the elimination of Cd from the soil [25]. The highly porous structure of BC increases the surface area [26], which could contribute to the adsorption of Cd through increased adsorption sites and surface complexation [27]. Due to ion exchange sites in clay minerals and organic matter, Cd cannot leach into groundwater or be absorbed by plants [28].

BC can impact the adsorption of Cd through the processes of chemical bonding, pH modification, and microbial activity, resulting in the formation of less toxic forms [29]. Learning complicated representations at different levels of abstraction is made possible by layer-wise layering of several nonlinear and linear computational modules [30]. Researchers have developed predictive algorithms that minimize errors in prediction versus experimental data for evaluating substance qualities [31]. Learning algorithms are developed using marked or unmarked data, or mixtures of data, to generate artificial intelligence algorithms. Data scientists utilize several machine-learning techniques for categorization and predictive modeling [32,33]. However, using quantitative analysis techniques to characterize soil’s properties and BC’s influence on Cd adsorption using advanced prediction methods is still unclear. Given the above problems, there is still a lack of quantitative and general guiding research, which limits the precise application of BC in improving HM-contaminated soil using advanced prediction models. 

To assess soil sensitivity, the research assembled representative soil samples across the country with an extensive range of physicochemical properties. The present study assessed the potential of Cd bioaccumulation in soils to evaluate the efficacy of several prediction models, including LSTM, BiGRU, and 5-layer CNN, for risk assessments. The objective was to provide an analytical approach to evaluating the impact of BC addition on Cd retention in soil characteristics, including pH, OC, CEC, EC, clay, and P. Additionally, AI algorithms were used to estimate the initial basic associations of soil parameters to enhance prediction models for BC-Cd soils and explore the contributing factors of Cd immobilization in soils. The analytical methodologies used in this investigation consisted of internationally recognized standards and innovative technologies that have emerged in recent years.

## 2. Materials and Methods

### 2.1. Soil Samples Description

This study was intended to select 44 soil samples from different areas of China (Table S1). The soil samples were collected from the upper layer of about 20 cm of the soil profile and brought to the Guangdong Academy of Agricultural Sciences. A composite sample was formed by thoroughly blending and homogenizing each subsample. After air-drying, a 2 mm sieve was used to collect debris from the composite soil sample [34].

### 2.2. Biochar Preparation

Rice straw biomass was purchased from the local commercial market and washed with distilled water (10–15 s) to remove dust particles. Subsequently, biomass was air-dried at room temperature and then oven-dried overnight with a constant hot air supply at 105 °C to remove moisture, after which it was mechanically ground. Rice straw BC at 450 °C was prepared with a retention time of 2 h. Afterward, it was cooled and passed through a 1 mm sieve [35]. The basic properties of BC are given in Table S2.

### 2.3. Incubation Experiment

An incubation experiment was conducted to evaluate the physicochemical characteristics of the soil by adding BC to enhance the adsorption of Cd. The significance of soil parameters and the sensitivity of BC-Cd adsorption were examined in various types of soils. Analytical-grade chemicals were employed. All chemical reagents were purchased from Sigma-Aldrich (Shanghai, China) and Sinopharm Chemical Reagent Company Ltd., Shanghai, China. De-ionized water (ultrapure) was used. Cd-contaminated soils were prepared artificially with Cd (NO_3_)_2_ (purity of 99.9%) aqueous solution (1000 mg/L). The purpose of soil spiking was to achieve a target concentration of about 3.3 mg/kg. The spiked soils were kept for 15 days to equilibrate Cd-contaminated soils. Afterwards, BC amendment was applied to soil at a rate of 1% (*w*/*w*). The moisture was maintained at 60–70%. The total duration of this experiment was 45 days. Before and after the incubation experiment’s completion, soil physicochemical analysis, which evaluated the pH (1:2.5 (*w*/*v*) using Mettler Toledo (Chengdu, China, model: Seven Compact 8210); the OC using the volumetric method (titration and colorimetric) by [36]; the EC (1:2.5 (*w*/*v*) using FOSS (model: TFS/YS-203) from Shanghai Hongyi Instrumentation Co., Ltd. (Shanghai, China); CEC using the ammonium acetate extracts method; and the total Cd (mg/kg) using Agilent Technologies (Chengdu, China) model: 7800 ICP-MS (inductively coupled plasma mass spectrometry), as well as tri acids (HNO_3_:H_2_SO_4_:HClO_4_) [2]. For soil texture, 40 mL of 1% sodium hexametaphosphate was applied to the soil (40 g). After waiting overnight, the soil was shifted to a dispersion cup (mechanical stirrer). The reading was taken using a Bouyoucos hydrometer. The texture of the soil was evaluated using the textural triangle. For diethylenetriaminepentaacetic acid (DPTA) analysis, the soil (8 g) was weighed, and an extraction solution of 16 mL of DTPA (pH 7.3) was added. After 60 min of shaking, the sample was filtered, and Cd was measured using ICP-MS [37].

### 2.4. Proposed Machine Learning Methods

#### 2.4.1. LSTM Model

**Figure 1:**
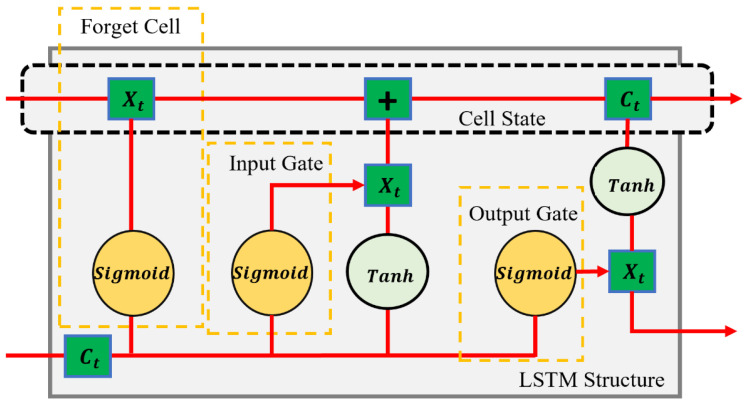
Long Short-Term Memory (LSTM).

LSTM models are specialized recurrent neural networks (RNN) capable of learning long-term dependencies in sequence data (Figure 1). Unlike standard feedforward neural networks, LSTMs have feedback connections that make them powerful for processing single data points and entire data sequences. A key feature of LSTM units is their ability to remember information for long periods, which is achieved through a complex mechanism of gates, including input, forget, and output gates. These gates effectively allow the network to add information to or remove it from the cell state, which is carefully regulated to prevent the vanishing gradient problem often encountered in traditional RNNs. LSTMs are widely used in various applications, such as time series prediction, natural language processing, and sequence generation tasks [38].

The LSTM model was applied to estimate the concentration of Cd (mg/kg) with various soil properties as input parameters. These properties included sand%, clay%, silt%, EC measured in µS/cm, pH, OC content in g/kg, CEC in cmol+/kg, and available P in mg/kg. The choice of LSTM for this task leverages its ability to process and learn from the sequential or structured nature of the input data, even though soil parameters are not sequential in the traditional sense. The model evaluated complex relationships and interactions between these soil properties to predict the Cd levels accurately. The sequential examination of various soil properties using LSTM has the potential to reveal patterns of Cd availability that are essential to environmental monitoring and agriculture. The LSTM’s architecture, including its memory cells and gates, can effectively learn from the intricacies of soil data, providing a powerful tool for predicting the HM contamination in soils.

#### 2.4.2. BiGRU Model

**Figure 2:**
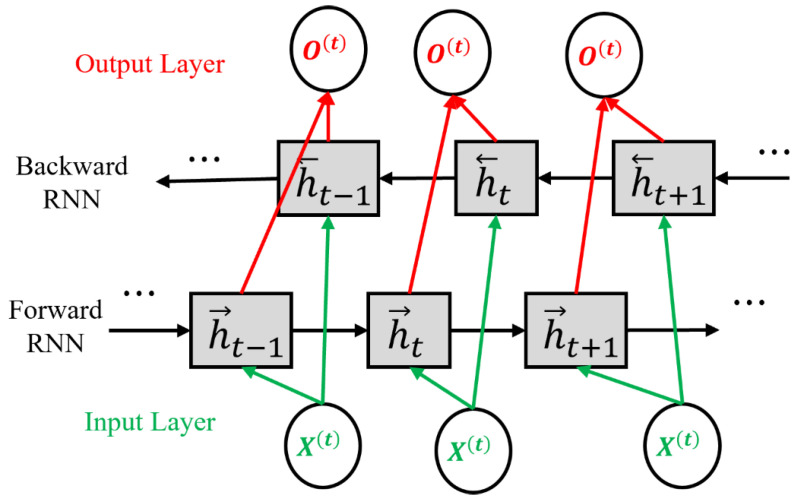
Bidirectional Gated Recurrent Unit (BiGRU).

In this research, BiGRU networks were employed to estimate Cd concentration based on a set of soil parameters. Figure 2 presents the block diagram of BiGRU. BiGRU models, an advancement of traditional GRU networks, process data in both forward and backward directions, allowing them to capture dependencies and patterns that might be missed when data are processed in a single direction. This dual-direction processing is particularly beneficial for understanding the complex interactions between soil properties and their impact on Cd availability. The study’s utilization of BiGRU networks enhanced the efficiency of parameterization and increased the ability to simulate the spatial and temporal relationships between BC-amended soils and Cd. Unlike LSTMs, GRUs simplify the gating mechanism without compromising the model’s ability to manage long-term dependencies. The bidirectional nature of BiGRUs offers a comprehensive perspective on the data, ensuring that the temporal dynamics and interdependencies of soil characteristics are thoroughly analyzed. This methodological approach underscores the potential of BiGRU models in environmental sciences, particularly for predicting HM concentrations in soils, thus providing valuable insights for soil management and remediation strategies. The use of BiGRU models highlights the innovative application of deep learning techniques in tackling complex environmental challenges [39].

#### 2.4.3. 5-Layer CNN

This study also explored CNN use by integrating a 5-layer CNN model equipped with max-pooling and fully connected (FC) layers. CNNs, known for image processing and machine learning, can computationally and adaptively learn spatial feature frameworks from the input for the purpose of analyzing complex, multivariate environmental data. The structure of the employed CNN model comprises five convolutional layers, each followed by a max pooling layer. Convolutional layers act as feature extractors from the input data, using learnable filters to identify and capture patterns such as edges, textures, or more complex features in deeper layers. After each convolutional operation, max-pooling layers are applied to reduce the dimensionality of the feature maps. This operation helps to make the representation smaller and more manageable and introduces translational invariance to the features, making the model more robust. The sequence of the convolutional and max pooling layers is designed to progressively refine the feature maps, ensuring that only the most relevant spatial features are retained and highlighted. A fully connected (FC) layer incorporates high-level, filtered data into the prediction model after decoding and merging layers are applied. The FC layer serves as a classifier, mapping the learned features to the output, which in this case is the estimated concentration of Cd [40].

**Figure 3:**
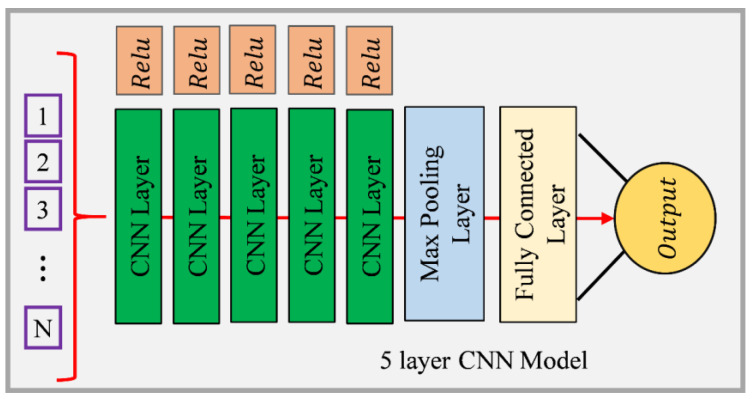
5-layer CNN model (Convolutional Neural Network).

Figure 3 shows the structure of the 5-layer CNN model. Applying a 5-layer CNN model to estimate Cd concentrations from soil parameters is innovative, as it transfers profound learning principles from their conventional domains to environmental science. By adapting CNN architectures, known for their efficiency in handling spatial data, to analyze and learn from soil property data, this study opens new pathways for advanced soil contamination analysis. The CNN model’s ability to discern intricate patterns within complex datasets could provide more accurate and reliable predictions of HM contamination, offering significant contributions to environmental monitoring and soil health assessment strategies.

### 2.5. Statistical Analysis

All data, including the soil’s physiochemical and amendment parameters, were derived from replications. A significance level of *p* < 0.05 was established for the descriptive data using a 95% confidence interval. The tables and graphical representation were generated using Sigma Plot 15.0, Microsoft Excel 2021, and Origin Pro 8.5. The experimental simulation for this study used AMD CPU Ryzen R7-4800H, along with an NVIDIA GeForce RTX 2060 6 GB GPU operating on Windows 10. The neural network model was developed using Python 3.8 and implemented using the Keras framework, with TensorFlow-GPU serving as the backend for deep learning computations.

## 3. Results and Discussion

### 3.1. Effect of the Amendment on Incubation Soil Physiochemical Characteristics Relates to Cd Concentrations

BC can immobilize HMs in soils, potentially aiding in the remediation of contaminated soils [41]. Solubility in the soil is attributed to precipitating, redox reactions, formation of complexes, and adsorption processes, which are facilitated by binding HMs to soil components and minerals [26]. The effect of BC additives on the physiochemical characteristics of incubated soils and Cd availability are detailed below.

#### 3.1.1. Soil pH Affects Cd Availability

**Figure 4:**
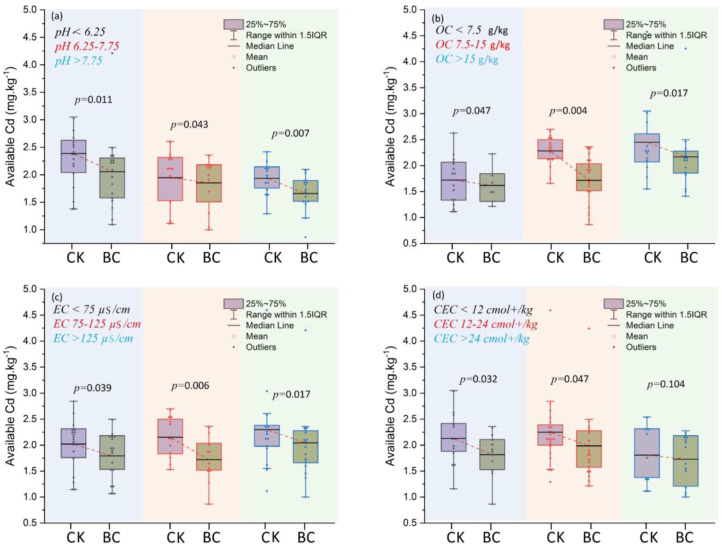
Effect of BC amendment on incubated soil physiochemical properties and Cd (mg/kg) availability. (**a**) pH, (**b**) OC, (**c**) EC, and (**d**) CEC.

The influence of applied BC on soil pH and Cd availability is given in Figure 4a. Soil pH was divided into three categories: (i) pH < 6.25, (ii) pH 6.25–7.75, and (iii) pH > 7.75. According to Figure 4a, the results indicated that the concentrations of available Cd were decreased in applied BC soils in all three categories of pH as compared to the control. The results revealed that applied amendment significantly decreased Cd availability in soils pH > 7.75 compared to pH < 6.25 and pH 6.25–7.75. Meanwhile, Figure 4a depicts that the highest Cd availability among control soils was observed at pH < 6.25. BC can alter several soil physicochemical properties owing to its varied composition and biodiversity activities [42]. BC usually has a pH range of 7–10, indicating an alkaline nature. BC is generated due to the pyrolysis of biomass, which has a higher pH than the original biomass [43]. Decontamination of HMs involves surface functional groups consisting of oxygen and the formation of complexes with BC, including electrostatic interactions, exchange of ions, and precipitation of chemicals [44]. Carbon sources, the formation of minerals, hydroxyl ion generation, and basic cation discharge may all be attributed to an increase in pH due to organic matter [10]. Reducing soil acidity and decelerating soil acidification are essential for preventing Cd accumulation in soil systems. Enhancing the buffering ability of soil pH can efficiently inhibit the process of soil acidification, diminish the concentration of accessible HMs in acidic soils, and hinder the soil–plant system absorption of HMs [45,46].

#### 3.1.2. Changing Soil Organic Carbon Affects Cd Availability

A comparison of BC amended and un-amended soils on organic carbon (OC) and Cd availability is given in Figure 4b. All incubated soils (amended and un-amended) were divided into three categories based on different ranges, such as low (OC < 7.5 g/kg), medium (OC 7.5–15 g/kg), and high (OC > 15 g/kg). The findings showed that adding BC to all soil samples significantly reduced the availability of Cd compared to the control. According to the findings, the lowest Cd availability was found in the OC (<7.5 g/kg) range of BC-amended soils compared to the control. The result indicated that the inclusion of BC systematically decreases the concentration of Cd in soils at each OC level compared to CK soils. BC minerals or surface groups illustrate inorganic components that mainly contribute to the adsorption of Cd. The findings indicated that the presence of BC can reduce the availability of Cd by binding to it or influencing the characteristics of the soil, thereby mitigating the impact of organic material solely on Cd mobility in the soil. The results demonstrated that using BC may enhance soil carbon sequestration and nutrient retention. It was shown that BC substantially increased the organic matter content, which was indirectly linked to changes in residual Cd concentration [47]. Organic matter is a significant component of soil fertility, providing nutrients for sustainable agriculture to maintain soil fertility. The OC level in the soil is essential for retaining nutrients and water, strengthening the soil structure, and supplying energy to soil microorganisms [48]. BC enhances soil fertility through the following mechanisms: retention of fertilizers, stimulation of microbial activity, carbon dioxide emission reduction, and immobilization of organic and inorganic pollutants [49]. The extensive integration of BC into soils would inevitably impact the increase in the quantity and composition of soil OC [50]. The effective sorption of HMs in contaminated effluents and soils has been associated with BC, which can potentially eradicate environmental contamination on a global scale [51].

BC possesses a substantial quantity of organic carbon, making it an exceptional asset in soil particle aggregation. Soil pollutants were immobilized by modifying soil physicochemical qualities, including carbon sequestration and fertility, with BC applied to the polluted soil [52]. The high area-to-volume ratio of these plants contributes to soil structure, porosity, water-holding capacity, aeration, nutrient availability, and buffering soil pH [53]. Moreover, they can retain plant nutrients, such as calcium, potassium, and phosphorus, reducing leaching [54]. Organic matter increases soil structure, porosity, and water-holding capacity, contributing significantly to climate change mitigation and carbon sequestration [55,56]. By providing nutrients to the soil, it promotes nutrient cycling through the action of soil microorganisms and serves as a source of nutrients. Additionally, it decomposes into stable aggregates that support soil structure, which supports soil porosity and improves soil structure [57].

#### 3.1.3. Soils EC Affects Cd Availability

The comparison of amended and un-amended soil EC (µS/cm) and Cd availability is shown in Figure 4c. EC (µS/cm) was divided into three different ranges: (i) EC < 75 µS/cm; (ii) EC 75–125 µS/cm; and (iii) EC > 125 µS/cm. Soil ecosystems are intricate, and the impact of added BC on soil EC-Cd availability can fluctuate based on various variables, including ecological factors, BC quantity, Cd concentration, and the characteristics of the soil. Figure 4c illustrates a particular set of scenarios in which the availability of Cd is substantially influenced by the increase in BC-added soil EC, but only in comparison to CK soils. The lowest Cd availability was found in EC (75–125 µS/cm) in BC-amended soil compared to the control. However, the results indicated that all unamended soils increased Cd availability with an increase in the EC range. EC is an indication of soil properties and also shows the soil water’s electrical carrying capacity. The low EC levels indicate limited nutrient availability and organic matter concentration. This finding further confirms the consistent relationship between the variance in BC and EC contents and the effect of Cd availability. The EC of soils has been shown to have a strong link with the movement of metals inside the soil. Soil salinization and acidity are prevalent and substantially affect Cd availability in soil systems [58,59]. BC played a crucial role in lowering the presence of Cd in the polluted soil. It achieved this by raising the soil’s pH, OC, EC, and P availability. Additionally, BC reduced the amount of Cd that can be easily exchanged in the soil by changing it into less accessible forms. As a result, there was a considerable decrease in Cd levels [60,61]. Excessive EC showed excess saline and inadequate nutrients, inhibiting development and appropriate plant growth. This may lead to soil–plant toxicity. As measured by EC, the ideal range for soil–plant nutrients is 0.2–1.2 (dS/m). However, adding BC may enhance soil EC due to the substantial amount of dissolved salts it contains. Recent research has shown that BC can increase the EC in soil [62].

#### 3.1.4. Changing Soil CEC Affects Cd Availability

A comparison of the effects of amended and un-amended soil CEC on Cd availability is presented in Figure 4d. Soil CEC (cmol+/kg) was also divided into 3 different ranges (i) CEC < 12 cmol+/kg; (ii) CEC 12–24 cmol+/kg; and (iii) CEC > 24 cmol+/kg. The results revealed that Cd availability decreased and was observed in all amended soils. The findings indicated that BC amendment significantly reduced Cd availability in soils compared to the control. The results showed that CEC indirectly correlated with Cd availability compared to the control. Application of BC led to enhancements in soil characteristics and resulted in the transformation of the Cd in the soil into more stable portions [63,64]. The rise in CEC may have led to the soil producing significant quantities of quinones, phenols, and carbonyls, thus resulting in greater adsorption of Cd [65,66].

The efficacy of immobilization is affected by several processes and circumstances, including precipitation, electrostatic contact, cation exchange, and complexation with functional groups [67,68]. A high CEC and a high adsorption capacity are both characteristics of BC which may have either negative or positive charges on its interface. The application of BC to enhance soil CEC resulted in a reduction in Cd through ion exchange and more efficient complexation adsorption into the soil [69]. Because soil clay particles assimilated more Cd as the CEC increases, the accessible Cd level in the soil was reduced significantly [70]. BC-amended soil exhibited an increase in CEC, which was dependent on the OC and clay along the surface area and electronegativity for ion adsorption sites capable of immobilizing Cd [71]. CEC was the most critical parameter significantly impacting Cd bioavailability and fractionation in soil [72].

#### 3.1.5. Effect of Soil Clay on Cd Availability

**Figure 5:**
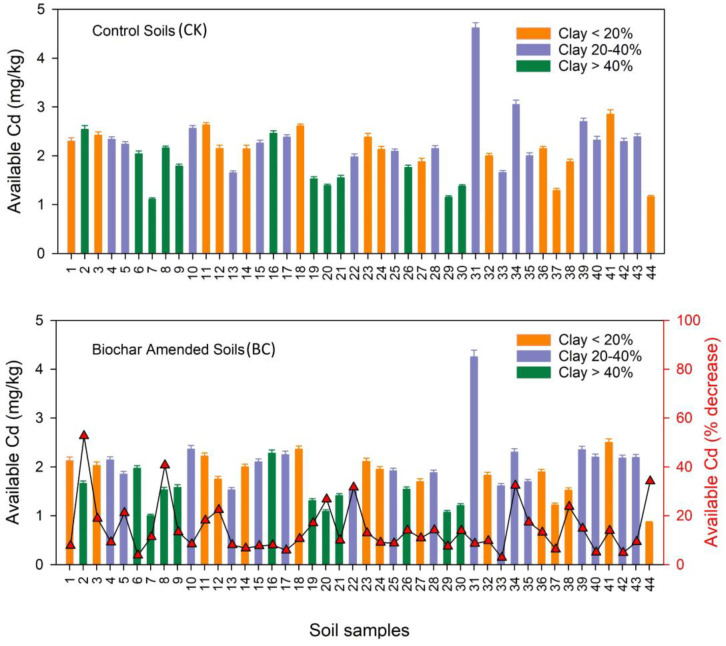
Impact of BC amendment on incubated soil clay and Cd (mg/kg) availability.

A comparison of amended and un-amended soil clay on Cd availability is shown in Figure 5. Soil clay was divided into three different categories: clay < 20%, clay 20–40%, and clay > 40%. According to Figure 5, the results showed that the BC amendment significantly decreased Cd availability compared to the control. The lowest Cd availability (almost 40–60% reduction) was found in high-clay-content, BC-amended soil compared to the control. However, the results indicated that all unamended soils had increased Cd availability along with the increased clay content. Minerals such as clay are formed from various elements, such as aluminum, silica, iron, etc. [73]. This finding further confirms the synergic relationship between BC and soil clay content compared to the control. The results revealed that soil clay was shown to have a strong link with the movement of Cd availability in the soil. The finding indicated that soil clay’s direct relationship with Cd availability and applied BC led to enhancements in soil characteristics and resulted in the immobilization of Cd in the soil. BC also reduced Cd availability by increasing pH, which increased the clay mineral surface negative charges and Cd ion adsorption [24]. Adding BC to medium or coarse-textured soils may improve the consistency of aggregate size and cationic retention.

Clay minerals and organic matter are critical components of soil that significantly impact the soil’s health, plant growth, and environmental sustainability [74,75]. Alumina, silica, and other elements constitute earth crust clays, and these soil metal sorbents reduce metal bioavailability [74]. Clay minerals’ exchangeable cations and anions contribute to their efficient contamination mitigation tools. Clays, which are negatively charged, absorb metal from soil solutions. Clay minerals use ion exchange, complexation, and direct bonding to absorb HMs [75]. Natural clay minerals have a strongly negatively charged layer to absorb cations. Minerals’ hydroxyl groups adsorb or combine HM to reduce its availability. In soil profiles, this adsorption reduces HM leaching. HM contaminants in soil have been successfully fixed in situ via adsorption and the formation of low-solubility HM precipitates using organic materials, including BC, clay minerals, and functional adsorbents [76]. Its considerable ecological and agronomic advantages render BC a highly prospective soil remediation agent, which has led to its widespread acceptance as a soil amendment.

### 3.2. Machine Learning-Based Soil Cd Concentration Prediction Models

The key goal of this research was to obtain representative soil samples from different regions of the country, each with distinct physical and chemical characteristics, to investigate the response of soils to the inclusion of BC and establish a prediction framework for the transformation and immobilization of Cd. The comprehensive analysis of the predictive modeling approaches was employed to estimate the concentration of Cd in soil based on an extensive dataset encompassing a wide array of soil properties, including sand%, clay%, silt%, EC, pH, OC, CEC, and available P. The significance of this study is underpinned by the environmental and agricultural importance of accurately assessing Cd levels in soils, given Cd’s known adverse effects on plant growth, soil health, and, ultimately, food safety. Cd is a considerable environmental concern due to its persistence and bioaccumulation in ecosystems, making its estimation in agricultural soils crucial for ensuring crop safety and ecological health. 

**Figure 6:**
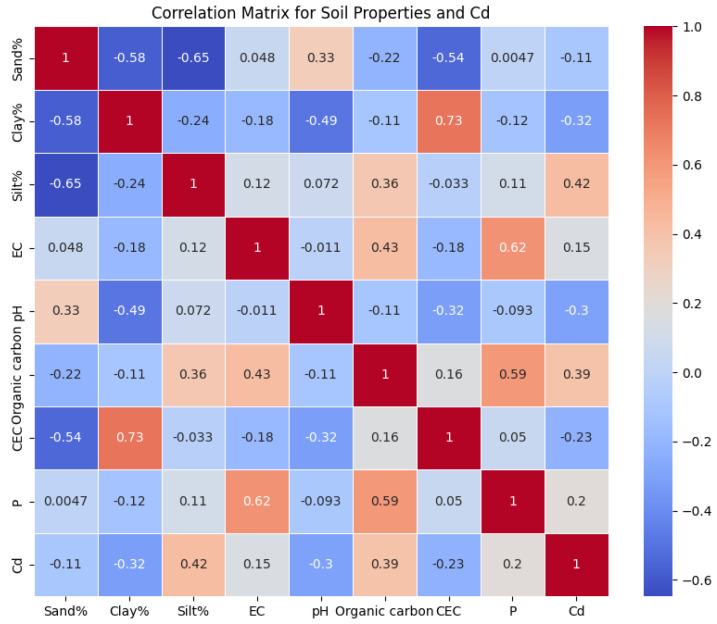
Correlation matrix for soil properties of CK.

**Figure 7:**
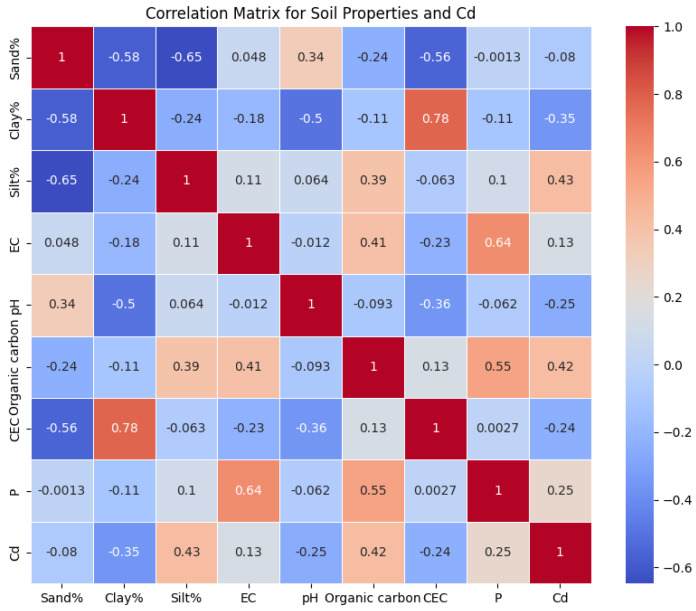
Correlation matrix of soil properties with BC.

The LSTM model was used to estimate the concentration of Cd from soil properties like sand%, clay%, silt%, EC, pH, OC content, CEC, and available P. The model learned from the sequential nature of input data, evaluating complex relationships between soil properties to predict Cd levels accurately. This sequential examination of soil properties can reveal patterns of Cd availability that are essential for environmental monitoring and agriculture. The LSTM’s architecture, including memory cells and gates, can effectively learn from soil data, making it a powerful tool for predicting HM contamination in soils. The research utilized BiGRU networks to estimate the concentration of Cd in soil parameters. BiGRU models process data in both forward and backward directions, capturing dependencies and patterns that might be missed when processed in a single direction. This dual-direction processing helps us to understand complex interactions between soil properties and their impact on Cd availability. BiGRU networks enhance parameterization efficiency and simulate spatial and temporal relationships between BC-amended soils and Cd. This methodological approach highlights the potential of BiGRU models in environmental sciences, particularly for predicting HM concentrations in soils, providing valuable insights for soil management and remediation strategies. The study used a 5-layer CNN model, which included max-pooling layers and a fully connected layer, to analyze complex environmental data. The model consisted of five convolutional layers, each with a max pooling layer, which extracted patterns from the input data. The model’s sequence refined the feature maps, ensuring that only relevant spatial features were retained. The fully connected layer incorporated high-level, filtered data into the prediction model, mapping learned features to the output, such as the estimated concentration of Cd in soil parameters. This innovative approach could contribute to environmental monitoring and soil health assessment strategies. The correlation heatmap was generated based on the dataset (Figure 6 and Figure 7). It provides a visual summary of the distributions of the soil properties and illustrates each feature’s spread and central tendency. The heatmap shows the correlation coefficients between all pairs of numerical variables, giving insight into the relationships between soil properties and Cd levels. In the case of CK soils, the findings showed positive relationships (Figure 6) between Cd content and silt% (0.42), organic carbon (0.39), EC (0.15), and P (0.20). The correlation matrices of the data of CK soils and Cd showed that there was a negative correlation with clay% (−0.32), sand% (−0.11), pH (−0.30), and CEC (−0.23). However, for soils modified with BC (Figure 7), the findings showed further evidence via negative relationships with sand% (−0.08), clay% (−0.35), pH (−0.25), and CEC (−0.24). The positive correlation matrices of BC-treated soils’ cumulative data revealed that the Cd levels were positively linked with the silt% (0.43), EC (0.13), OC (0.42), and P (0.25) [77]. The comprehensive details are given in Section 3.1.

#### 3.2.1. Case 1: Assess CK Soils Using Machine Learning Models

**Figure 8:**
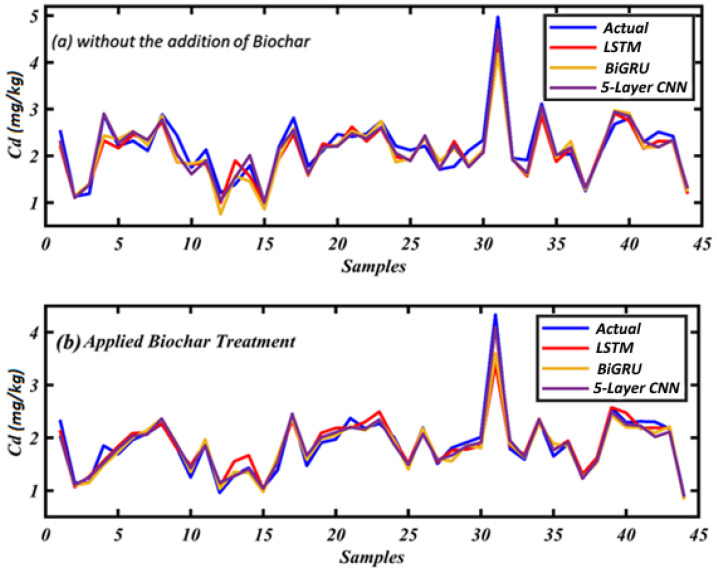
The effectiveness of machine learning models in estimating Cd.

Figure 8a compares the actual Cd concentrations against the predictions made by three machine learning models. This comparison is made in the context of soil samples without adding BC. The BiGRU model showed commendable performance, with an $R^{2}$ value of 0.8537, indicating that the model’s predictions could explain approximately 85.37% of the variance in the actual Cd data. The model’s mean absolute error (MAE) was reported at 0.1986, and the mean squared error (MSE) was 0.0650, with a root mean square error (RMSE) of 0.2550. These metrics suggest that the BiGRU model predictions were relatively close to the actual values, with a moderate spread of errors. The LSTM model, which also considered sequence data, yielded slightly less accurate results, with an $R^{2}$ value of 0.8473. The MAE for this model was 0.2127, the MSE was 0.0678, and the RMSE was 0.2605, which are all slightly higher than those for the BiGRU model, indicating a lower performance in terms of capturing the variation in Cd concentrations. The 5-layer CNN model, traditionally used in image processing but adapted here for sequence prediction, outperformed both recurrent neural network models with an $R^{2}$ value of 0.9107, showing that over 91% of the variability in the actual Cd levels could be accounted for by the CNN model’s predictions. Additionally, this model achieved the lowest MAE of 0.1602, the lowest MSE of 0.0439, and the lowest RMSE of 0.2121, indicating the highest accuracy and precision among the three models.

Overall, Figure 8a demonstrates that, while all models were relatively proficient in estimating Cd concentrations, the 5-layer CNN model appeared to be more effective in this context. This might be due to its superior ability to capture the complex non-linear relationships between the soil properties and the Cd levels without the influence of BC. The success of the CNN model here suggests that it may be a promising tool for environmental monitoring applications where understanding the baseline contamination levels is crucial.

#### 3.2.2. Case 2: Evaluated Applied BC Soils Using Machine Learning Models

Figure 8b showcases the effectiveness of machine learning models in estimating Cd concentrations in soil, considering the addition of BC. BC, an amendment known for its ability to absorb and immobilize HMs, provides a unique context for the machine learning models to capture the altered relationships between soil properties and Cd availability. The BiGRU model demonstrated a strong correlation between predicted and actual values, with an $R^{2}$ value of 0.9321, indicating that the model explained 93.21% of the variance in Cd concentrations. The model improved its predictive accuracy in the presence of BC, with an MAE of 0.1270, an MSE of 0.0165, and an RMSE of 0.1286, all lower than the values reported in Case 1. Similarly, the LSTM model showed a notable increase in performance with BC-treated soil data, yielding an MAE of 0.1105, an MSE of 0.0161, and an RMSE of 0.1256. The $R^{2}$ value for this model stood at a robust 0.9413, reflecting its enhanced ability to predict Cd levels with BC incorporation. Outperforming both recurrent models, the 5-layer CNN model attained the highest precision, with an $R^{2}$ value of 0.9558, suggesting that 95.58% of the variance in the actual Cd data could be explained by the CNN model’s predictions in BC-amended soils. The model registered the lowest error metrics, with an MAE of 0.0983, an MSE of 0.0145, and an RMSE of 0.1206, underlining its superior predictive power in this scenario.

The results in Figure 8b reinforce the potential of advanced deep learning models in environmental applications, especially for assessing the impacts of soil amendments such as BC on HM contamination. The improved accuracy and lower error rates across all models with the addition of BC suggest that these models can capture the complex interactions that BC induces in the soil matrix, subsequently affecting Cd bioavailability. These insights are valuable for designing strategies to mitigate soil contamination and evaluating BC’s effectiveness as a remediation tool. The superior performance of the 5-layer CNN model, in particular, highlights the adaptability and robustness of convolutional networks in handling diverse and complex environmental datasets.

## 4. Conclusions

The study compared the effectiveness of deep learning machines like LSTM, BiGRU, and 5-layer CNN in forecasting Cd concentrations. A significant negative association between the two variables was found, mainly due to the impact of BC on soil characteristics and Cd immobilization. BC effectively mitigates the presence of HMs in soil and inhibits their penetration into the soil system. The BiGRU model explained 93.21% of the Cd concentration variance after adding BC, while the LSTM model improved with BC-treated soil data. The 5-layer CNN model predicted 95.58% of the variance in Cd data in BC-amended soils. The study highlights the significance of machine learning models that can predict the impact of BC amendments on soil’s chemical and physical properties, such as pH, OC, clay minerals, and Cd availability. Machine learning models are capable of generating precise assessments of soil Cd content and predicting the probability of soil Cd transformation and immobilization. These data could establish sustainable soil management and environmental protection strategies. The study proposes the development of ecological soil remediation systems to efficiently address heavy metal contamination in soils, with the goal of promoting environmental sustainability.

## Data Availability

The data presented in this study are available on request from the corresponding author. The data are not publicly available due to privacy.

## Supplementary Materials

The following supporting information can be downloaded at: https://www.mdpi.com/article/10.3390/toxics12080535/s1, Table S1: Area description of the soil samples collected; Table S2: Rice straw biochar properties; Table S3. Comparison with different studies.
