# Supplementary material for: Quantitative Soil Characterization for Biochar–Cd Adsorption: Machine Learning Prediction Models for Cd Transformation and Immobilization
Source: Toxics. 2024 Jul 24;12(8):535. doi: 10.3390/toxics12080535 (PMC11359006; doi:10.3390/toxics12080535)
Supplement: Supplementary file 1 [file toxics-12-00535-s001.zip › toxics-3088314-supplementary.pdf]

Quantitative soil characterization for biochar–Cd adsorption: Machine learning prediction models for Cd transformation and immobilization

Muhammad Saqib Rashid[1], Yanhong Wang[1], Yilong Yin[1], Balal Yousaf[2], Shaojun Jiang[1], Adeel Feroz Mirza[3], Bing Chen[4], Xiang Li[1*], Zhongzhen Liu[1*]

[1]*Key Laboratory of Plant Nutrition and Fertilizer in South Region, Ministry of Agriculture, Guangdong Key Laboratory of Nutrient Cycling and Farmland Conservation, Institute of Agricultural Resources and Environment, Guangdong Academy of Agricultural Sciences, Guangzhou, Guangdong 510640, China*

[2]*Department of Technologies and Installations for Waste Management, Faculty of Energy and Environmental Engineering, Silesian University of Technology, 44-100 Gliwice, Poland*

[3]*Department of Mechanical and Energy Engineering, Southern University of Science and Technology, China*

[4]*Institute of Animal Science, Guangdong Academy of Agricultural Sciences, Collaborative Innovation Center of Aquatic Sciences, Key Laboratory of Animal Nutrition and Feed Science in South China, Ministry of Agriculture and Rural Affairs, Guangdong Provincial Key Laboratory of Animal Breeding and Nutrition, Guangzhou 510640, China.*

---

* Corresponding author: Zhongzhen Liu，Xiang Li

E-mail address: lzzgz2001@163.com, lixiang142213@163.com

Table S1: Area description of the soil samples collected.

Sr.#	City	Province	Texture
1	Hailun	Heilongjiang	Loam
2	Guiyang	Guizhou	Clay
3	Zhengzhou	Henan	Silt loam
4	Nanjing	Jiangsu	Silty clay loam
5	Quzhou	Zhejiang	Silty clay
6	Nanchang	Jiangxi	Silty clay
7	Zhanjiang	Guangdong	Clay
8	Danzhou	Hainan	Clay
9	Zhanjiang	Guangdong	Clay
10	Shengyang	Liangning	Silty clay loam
11	Quzhou	Zhejiang	Loamy sand
12	Zhengzhou	Henan	Silt loam
13	Baise	GuangXi	Clay loam
14	Dalian	Liaoning	Loam
15	Harbin	Heilongjiang	Silty clay loam
16	Yueyang	Hunan	Silty clay
17	Guangzhou	Guangdong	Loam
18	Zhuzhou	Hunan	Sandy loam
19	Nanchang	Jiangxi	Clay
20	Zhanjiang	Guangdong	Clay
21	Zhanjiang	Guangdong	Clay
22	Sanya	Hainan	Sandy clay loam
23	Sanya	Hainan	Loam
24	Sanya	Hainan	Sandy loam
25	Panzhihua	Sichuan	Sandy clay loam
26	Panzhihua	Sichuan	Clay
27	Panzhihua	Sichuan	Loamy sand
28	Wuhu	Anhui	Clay loam
29	Guiyang	Guizhou	Clay

30	Guiyang	Guizhou	Clay
31	Hangzhou	Zhejiang	Silt loam
32	Hefei	Anhui	Loam
33	Hefei	Anhui	Loam
34	Chengdu	Sichuan	Silt loam
35	Jiangyou	Sichuan	Loam
36	Mianyang	Sichuan	Silt loam
37	Yibin	Sichuan	Loamy sand
38	Zhenjiang	Jiangsu	Silt loam
39	Changchun	Jilin	Silt loam
40	Shenyang	Liaoning	Silty clay loam
41	Changchun	Jilin	Silt loam
42	Yanbian Chaoxian	Jilin	Silty clay loam
43	Shenyang	Liaoning	Silty clay loam
44	Changsha	Hunan	Silt loam

Table S2: Rice straw biochar properties.

	pH	N%	H%	OC (g/kg)	Cd (mg/kg)	P (g/kg)	CEC (cmol+/ kg)	EC (dS/m)	Average pore volume (cm^3/g)	S_{BET} (m^2/g)	Average pore size (nm)
BC	7.24	0.55	3.36	14.77	0.08	2.20	67	2.17	0.07	11.09	20.95

Table S3. Comparison with different studies.

Prediction models	Pollutants	Prediction accuracy	Reference
CNN	Cd, Pb, Cr, As, and Hg	R^2: 0.81, 0.70, 0.68, 0.462, and 0.816, respectively	[1]
CNN and GRU	$PM_{2.5}$	R^2: 0.76 and 0.70, respectively	[2]
BiGRU, LSTM, and 5 Layer CNN model	Cd	R^2: 0.85, 0.84, and 0.91, respectively	Our study

Note: convolutional neural network: CNN; Gated recurrent unit: GRU; Bidirectional Gated Recurrent Unit: BiGRU; Long Short-Term Memory: LSTM.

References:

1. Li, P.; Hao, H.; Mao, X.; Xu, J.; Lv, Y.; Chen, W.; Ge, D.; Zhang, Z. Convolutional Neural Network-Based Applied Research on the Enrichment of Heavy Metals in the Soil–Rice System in China. *Environ. Sci. Pollut. Res.* **2022**, *29*, 53642–53655, doi:10.1007/s11356-022-19640-x.
2. Xie, H.; Ji, L.; Wang, Q.; Jia, Z. Research of PM2.5 Prediction System Based on CNNs-GRU in Wuxi Urban Area. *IOP Conf. Ser. Earth Environ. Sci.* **2019**, *300*, 032073, doi:10.1088/1755-1315/300/3/032073.
